# Does asset ownership influence sexual risk-taking behaviors among women engaged in sex work in Southern Uganda? A mediation analysis

**DOI:** 10.1186/s12905-022-02129-7

**Published:** 2022-12-22

**Authors:** Josephine Nabayinda, Joshua Kiyingi, Samuel Kizito, Edward Nsubuga, Proscovia Nabunya, Ozge Sensoy Bahar, Natasja Magorokosho, Jennifer Nattabi, Susan Witte, M. Ssewamala Fred

**Affiliations:** 1 International Center for Child Health and Development (ICHAD), Washington University in St. Louis Brown School, 1 Brookings Drive, St. Louis, MO 63130 USA; 2 Columbia University School of Social Work, 1255 Amsterdam Avenue, New York, NY 10027 USA

**Keywords:** Assets/economic resources, Sexual-risk-taking behaviors, Women engaged in sex work, Poverty

## Abstract

**Background:**

Economic vulnerability influences women engaged in commercial sex work (WESW) to further engage in sexual risk behaviors, as they often have multiple customers and engage in unprotected sex for financial gains. This study examined asset ownership’s direct and indirect impact on sexual risk-taking behaviors among WESW in Southern Uganda, a very vulnerable group of women at high risk for contracting HIV and other STIs.

**Methodology:**

We used baseline data from the *Kyaterekera* study, an NIH-funded study among WESW aged 18–55 across 19 HIV hotspots in Southern Uganda. Structural equation modeling was used to examine the direct, indirect, and total effects of assets—defined as ownership of physical and financial resources—on sexual risk-taking behaviors among WESW.

**Results:**

Results showed that asset ownership was associated with a decrease in depression (β =  − 0.096 [95% CI − 0.191, − 0.001], *p* = 0.050) and increased access to medical care (β = 0.174 [95% CI 0.072, 0.275], *p* = 0.001).We also found that an increase in access to medical care was associated with decreased sexual risk-taking behaviors (β =  − 0.107 [95% CI − 0.210, − 0.004], *p* = 0.041). We observed a specific indirect effect between assets and sexual risk-taking behaviors through access to medical care (β =  − 0.019 [95% CI − 0.040, − 0.002], *p* = 0.05). Mediation contributed 31% of the total effects of asset ownership on sexual risk-taking behaviors.

**Conclusion:**

To our knowledge, this is among the few studies to examine the impact of asset ownership on sexual risk-taking behaviors among WESW in Southern Uganda**.** Findings from this study indicate that increasing access to economic resources may reduce the risk of WESW engaging in unprotected sex for higher income, which limits the spread of HIV among this population. The results also indicate that asset ownership may allow women to access healthcare services.

## Introduction

Women engaged in sex work (WESW) are at a higher risk of acquiring HIV compared to other women [1]. The current statistics indicate that, in sub-Saharan Africa, the risk of HIV is 26 times higher among WESW than in other women [2], while in Uganda—the location of the current study—the HIV prevalence among WESW is 31.3%, which is six times higher than the national average [3]. According to the *Consolidation and Building Consensus* report, over 130,000 women are engaged in sex work in Uganda [4]. The elevated risk for HIV contraction among WESW has been linked to economic vulnerability, driven by factors such as a lack of skills for formal employment due to low levels of education [5]. Economic deprivation further increases the risk of engaging in sexual risk-taking behaviors among WESW. For instance, the need for an extra pay may influence WESW to engage in unprotected sex [6]. Despard and colleagues highlighted that financial shocks among low and middle income households increases the risk for material hardships, and makes it hard for the families to fulfill the basic needs of life [7]. Hence, economic stability in the form of owning assets and having a reliable source of income helps households to meet their financial needs such as providing housing, food and other household expenses[7]. For WESW, the limited choices for other means of survival pushes some of them to engage in more risk-taking behaviors, which increases their risk of contracting HIV/AIDS [8].

Furthermore, poverty makes it hard for WESW to access HIV prevention and treatment services due to the associated costs [9]. Poverty also increases women’s vulnerability to other life stressors such as depression and intimate partner violence, which are significant risk factors for risky sexual behaviors, such as losing the motivation to protect themselves when having sex [10, 11]. The Ford Foundation suggests that placing assets in people's hands, especially low-income earners, gives them the independence they need to resist risks and the power to pursue productive livelihoods [12]. On the other hand, the NSWP report denotes that, increasing economic options for WESW enables them to receive greater financial security, which impacts their decisions in shaping their lives [13] .

Several interventions have been designed with specific attention to improving the welfare of WESW. However, most of these interventions have focused on reducing stigma, discrimination, promoting access to safer sex and eliminating violence against WESW by their clients and the police [14], with less attention to the broader economic needs of WESW. Yet, according to Moret, providing economic resources to WESW helps them decline their client’s requests for unprotected sex, which enhances their ability to negotiate for safer sex [15]. Studies that have incorporated economic empowerment interventions among WESW, have indicated the ability of such interventions to promote financial security, which reduces the women’s vulnerability to unprotected sex and other HIV risk behaviors. For instance, a study conducted among female sex workers (FSW) in Tanzania revealed that, providing safety nets for times of financial needs allowed FSW to create greater financial security, resulting in their ability to negotiate condom use [16]. Similarly, Odek and colleagues conducted a study on the effects of micro-enterprises services on HIV risk behavior among female sex workers in the slums of Kenya. Results from their study revealed a reduction in the number of sexual partners reported by FSW [17]. However, none of these studies examined the pathways through which economic empowerment reduced the sexual risk-taking behaviors in this population.

In Uganda, there is dearth of literature on the role of economic resources in reducing sexual risk behaviors among WESW. Most of the studies in the country that employ economic strengthening interventions have focused on adolescents [18–21], with less attention to WESW. Nonetheless, findings from these studies show that adolescents who had access to economic resources were less approving of sexual risk-taking behaviors [18, 20]. To our knowledge, no studies have been conducted in the country to examine the impact of economic resources on risky sexual behaviors among WESW, and the pathways through which this effect is achieved. Therefore, extending access to economic resources among WESW, offers a way out of poverty, which women can use to generate economic, psychological, and social benefits that foster resilience to their engagement in risk behaviors [22]. This study uniquely examines how asset ownership reduces the likelihood of engaging in other sexual risk-taking behaviors, including unprotected sex, and number of sexual partners among WESW. This population is at higher risk of acquiring HIV and other STIs, due to their use of multiple sexual partners and having unprotected sex [1]. We hypothesize that WESW with more assets are less likely to engage in other risky sexual behaviors compared to those with fewer assets.

### Theoretical framework

This study is guided by both the resilience and asset theories [23]. Both theories have been used in previous studies that examined the association between economic resources and sexual risk behaviors in low-resource communities [20, 21]. The resilience theory identifies an individual with the ability to adapt successfully and bounce back from risks and adversities through the provision of resources [24]. Studies employing the resilience theory suggest that resources such as cash transfers and other assets could help individuals overcome the consequences of risk exposure and the adverse outcomes associated with risk behaviors [20, 25]. Similarly, the asset theory posits that when individuals own economic assets, they may yield multiple positive outcomes, including increased economic stability and socio-behavioral and psychological benefits [23]. For instance, a study conducted by Witte et al. on the efficacy of a savings-led microfinance intervention among WESW in Mongolia indicated that a matched savings program for asset building was successful in reducing sexual risks among WESW [6]. Sherraden adds that people will think and behave differently when they accumulate assets, but also the world will respond to them differently [23]. Therefore, ownership and control of assets such as land and housing offer considerable benefits to individuals, such as having a positive attitude towards the future, which provides a foundation for minimizing risk-taking and increasing individual self-efficacy. Velez-Grau and colleagues conducted a study in Kazakhstan to examine the role of microfinance in preventing HIV among women who use drugs and engage in sex work. Findings revealed that, women who received a microfinance component had the capacity to plan for their future and the motivation to seek alternative sources of income [26]. Therefore, for WESW, providing assets could help in responding to the financial setbacks they face, that forces them to engage in risky behaviors in search for an income. Therefore, we hypothesize that ownership of assets could reduce sexual risk behaviors among WESW by building resilience to risky situations and standing economic shocks. We further hypothesize that this effect is not only direct, but can be mediated through other factors, such as reducing depression, and increasing access to medical care. Our study focuses on economic assets, including but not limited to financial assets such as cash, land, livestock, housing, business assets, and any other physical assets such as vehicles and communication devices.

## Methods

### Study design

We used baseline data from a randomized controlled study funded by the National Institute of Mental Health (NIMH). The study aimed to assess the efficacy of a combination intervention when added to the traditional HIV risk reduction (HIVRR) in reducing the incidence of STI and HIV among WESW in Southern Uganda. Between June 2019 and March 2020, the study team employed a randomized cluster design to recruit 542 WESW from 19 HIV hotspots (areas that are reported to have high HIV prevalence rates) in the region. According to Grabowski et al., HIV hotspots serve as drivers of transmission to the neighboring areas of lower prevalence [27]. Women were eligible to participate in the study if they fulfilled the following inclusion criteria: 1) at least 18 years of age, 2) engaged in transactional sex, defined as having vaginal or anal sexual intercourse in exchange for money, alcohol, or other goods in the last 30 days, and 3) engaged in at least one episode of unprotected sexual intercourse in the preceding 30 days.

### Study setting

As of December 2020, the estimated total number of people living with HIV in Uganda was 1.4 million, with a prevalence rate of 31.3% among WESW, a percentage higher than the national prevalence of 5.4% [3]. The HIV prevalence in Masaka region where the study was conducted, is 11.7%, which is twice higher than the national average [3]. In addition, there is an estimated total of 1,332 WESW within the study target hotspots [28]. For this study, the research team engaged community stakeholders to identify and recruit WESW into the study. Details of the study’s methodology are described in our published protocol [29].

### Ethics and informed consent

The study received approval from the Washington University in St. Louis Institutional Review Board (IRB #201811106), Columbia University IRB (IRB #AAAR9804), the Uganda Virus Research Institute (UVRI #GC/127/18/10/690), and the Uganda National Council of Science and Technology (UNCST #SS4828). All methods were performed in accordance with the relevant guidelines and regulations, and all women in the study provided voluntary written informed consent prior to participating in the study. All the research assistants received ethics training in the form of Collaborative Institutional Training Initiative (CITI) Human Subjects training and Good Clinical Practice training. The interviews were conducted in a secluded area to ensure privacy and to avoid interference. All consent forms and related study materials, including data collection measures, were translated into Luganda by a certified translator from the School of Languages, Literature, and Communication at Makerere University, then reviewed by a team of stakeholders and research staff to ensure that they are culturally and linguistically appropriate [30].

### Study measures

#### Outcome

The primary outcome of this study was sexual risk-taking behaviors, measured using four items adapted from Schilling et al. [31]. Items included 1) the total number of customers that the participant had sex within the past 30 days, 2) the number of times in total a participant had vaginal sex with any of the customers in the past 30 days, 3) the number of times a condom was used during these encounters, and 4) whether participants could accept more money, goods or extra services from their paying customers for unprotected sex. A latent variable for sexual risk-taking behaviors was generated from the four questions.

#### Independent variable

Asset ownership was defined in terms of ownership of financial and physical resources. It was measured using the asset index, a scale comprising 21 items that assessed whether the women owned various items such as land, gardens, house, rental property, a small business, car, cell phone, and cattle, to mention a few. We coded each item with a "1" if the participant owned the item and a "0" if the participant did not. We used principal components analysis (PCA) to generate a latent factor variable for asset ownership.

#### Mediator variables

Mediator variables included depression and access to healthcare. Depression was measured using a 6-item scale from the Brief Symptoms Survey [32]. The 6-items assessed whether respondents had experienced any of the depressive symptoms, including 1) thoughts of ending life, 2) feeling lonely, 3) feeling sad, 4) feeling no interest in things, 5) feeling hopelessness about the future, and 6) feelings of worthlessness. Responses were coded from 1 = “Not at all,” 2 = “A little bit,” 3 = “Moderately,” 4 = “Quite a bit,” and 5 = “Extremely.” The theoretical range was 0–24 (alpha = 0.82). Using PCA, we extracted one factor that we used to measure depression.

Access to health services was measured using a 6-item scale related to seeking medical care in the past 12 months [33]. Responses were rated on a 5-point scale with 1 = “Strongly Agree,” 2 = “Somewhat Agree,” 3 = “Uncertain,” 4 = “Somewhat Disagree,” and 5 = “Strongly Disagree.” The six items included: a) “If I need medical care, I can get admitted without any trouble,” b) “It is hard for me to get medical care in an emergency,” c) “Sometimes I go without the medical care I need because it is too expensive,” d) “I have easy access to the medical specialists that I need,” e) “Places, where I can get medical care, are very conveniently located,” and f) “I am able to get medical care whenever I need it.” Two items were captured in the inverse direction and had to be reverse coded to match the other items. We used PCA to generate a latent variable for access to health care in the SEM.

Participants' age and education level were included in the model as control variables.

### Data analysis

We conducted the descriptive analysis using STATA SE, Version 17 (StataCorp, college station, Texas 77845). We declared data to be survey data to adjust for clustering at the level of the HIV hotspots. Continuous variables were summarized using means and standard deviations. Categorical variables were summarized using percentages. We used SPSS and MPlus version 8.1 (Muthen and Muthen) to fit the principal component analysis (PCA) and the structural equation models (SEM), respectively, that assessed the effects of asset ownership on sexual risk-taking behaviors among the WESW. Prior to performing PCA, we assessed the data for suitability of PCA using the KMO test for sampling adequacy, which yielded a value of 0.898 (critical value 0.5). We also computed the Bartlett’s test for sphericity, where we aimed for a significant *p* value. The corresponding *p* value was < 0.001.

The results tables reported both the non-standardized coefficients (B) and the standardized coefficients (β) and their 95% confidence intervals. For all the analyses, the level of significance was set at 0.05.

To test for the goodness of fit of the final model, we used several parameters, including 1.) the chi-square for the goodness of fit, where we aimed for a non-significant *p*-value. 2.) Root Mean Square Error of Approximation (RMSEA) less than 0.063) Standardized Root Mean Square Residual (SRMR) less than 0.08, and 4.) Comparative Fit Index (CFI) of at least 0.95. We estimated the direct effects, indirect effects through the mediators, and total effects of asset ownership on sexual risk-taking behaviors. The direct effect is the effect of asset ownership on sexual risk-taking behavior in the absence of mediators. A specific indirect effect represents how much that mediator explains the asset ownership effect. The sum of the individual-specific indirect effects constituted the total indirect effects. The total effect is the sum of the direct and indirect effects. Using the indirect and total effects, we determined the proportion of the mediated effect.

## Results

Sample characteristics are presented in Table 1. All 542 participants were included in the analysis. The average age was 31.4 years (SD = 7.18). Most participants (61%) were either divorced/widowed or separated from their partners, and 64% had achieved up to primary education. In addition, 54% of the women lived in small towns. About a quarter (24%) of participants reported working for pay at baseline. Women reported an average score of 5.52 (SD = 4.14) on asset ownership and a mean = 10.91 (SD = 4.98) on the depression scale. The overall mean score for access to medical services was 16.7 (SD = 4.5).

**Table 1:** Sample characteristics (N = 542)

Variables	Number (%)/SD
Age (Mean, SD)	31.4 (7.18)
Marital status	
Single/never married	72 (13.3)
Married/in a relationship	139 (25.7)
Divorced/widowed/separated	331 (61.1)
Education level	
Up to primary education	344 (63.47)
Secondary and higher education	198 (36.53)
Site location	
Fishing sites	131 (24.2)
Rural	120 (22.1)
Small towns	291 (53.7)
Currently working for pay	128 (23.6)
Access to medical services (mean, SD)	19.22 (4.51)
Asset ownership (mean, SD)	5.52 (4.14)

### Mediation analysis

**Fig. 1:**
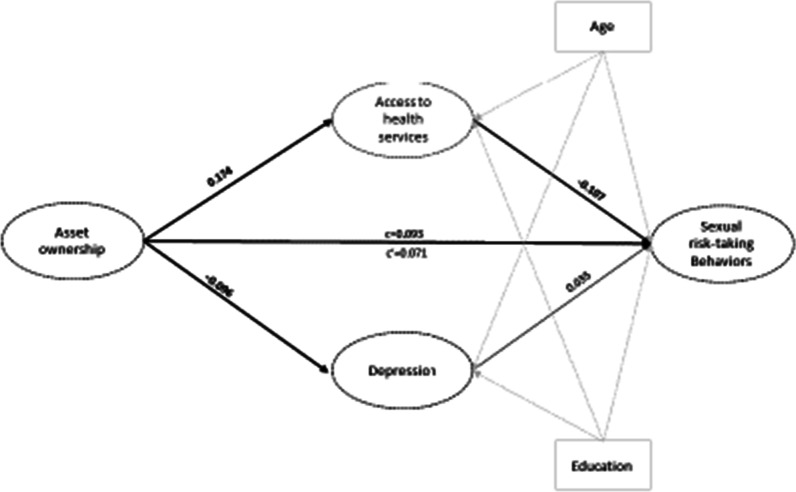
Structural equation model showing the effect of asset ownership on sexual risk taking behaviors among WESW in Southern Uganda. We reported standardized coefficients. Significant associations are illustrated with bold arrows

Table 2 and Fig. 1 present results from the structural equation modeling analysis for the mediators of the effects of assets on sexual risk-taking behavior. The results show that asset ownership was significantly associated with a decrease in depression (β =  − 0.096 [95% CI − 0.191, − 0.001], *p* = 0.050). There was also a statistically significant association between asset ownership and access to medical care. Specifically, increase in asset ownership was associated with increased access to medical care (β = 0.174 [95% CI 0.072, 0.275], *p* = 0.001). The increased access to medical care was associated with decreased sexual risk-taking behaviors (β =  − 0.107 [95% CI − 0.210, − 0.004], *p* = 0.041). We observed a specific indirect effect between asset ownership and sexual risk-taking behaviors through access to medical care (β =  − 0.019 [95% CI − 0.040, − 0.002], *p* = 0.05). Both the direct (β = 0.093 [95% CI: 0.003, 0.183], *p* = 0.044) and total indirect effects (β =  − 0.022 [95% CI − 0.044, − 0.001], *p* = 0.046) were statistically significant. However, the total effect was not significant. Overall, the mediation contributed 31.0% of the total effects of asset ownership in reducing sexual risk-taking behaviors among WESW. The model fitness parameters included CFI = 0.934, RMSEA = 0.058, and SRMR = 0.049.

**Table 2:** Mediation analysis between asset ownership and sexual risk taking behaviors

Variable	B	β (95% CI)	*p*-value
Asset ownership on mediators			
Depression	− **0.140**	− **0.096 (**− **0.191–** − **0.001)**	**0.050**
Access to health care	**0.210**	**0.174 (0.072–0.275)**	**0.001**
Mediators on sexual risk-taking behaviors			
Depression	3.521	0.035 (− 0.062–0.131)	0.478
Access to health care	− **13.140**	− **0.107 (**− **0.210–** − **0.004)**	**0.041**
Specific indirect effects			
Depression	− 0.494	− 0.003 (− 0.013–0.007)	0.507
Access to health care	− **2.761**	− **0.019 (**− **0.040–** − **0.002)**	**0.05**
Effects			
Direct effect	**13.756**	**0.093 (0.003–0.183)**	**0.044**
Total indirect effect	− **3.255**	− **0.022 (**− **0.044–** − **0.001)**	**0.046**
Total effect	10.501	0.071 (− 0.018–0.160)	0.117
Effect mediated		31.0%	
Model fitness			
Chi-Square		χ^2^ (239) = 672.06, *p* < 0.001	
RMSEA		0.058	
CF1		0.934	
TLI		0.924	
SRMR		0.049	

Text in bold shows statistically significant associations

## Discussion

The study examined the role of asset ownership in influencing sexual risk-taking behaviors among WESW in Southern Uganda. We hypothesized that WESW with more assets are less likely to engage in other risky sexual behaviors compared to those with fewer assets. This study's findings suggested a direct association between asset ownership and sexual risk-taking behaviors. These results support the theories used in this study that assets are protective against high-risk taking sexual behaviors [23, 24]. Studies conducted in Uganda have documented a strong relationship between economic resources as a strategy for minimizing the chances of engaging in risky behaviors among women. To illustrate, Ssewamala and colleagues examined the effect of asset ownership on sexual risk-taking intentions among orphaned female adolescents. Results from their study indicated that the adolescents who participated in the intervention reported reduced sexual risk-taking intentions [20]. Similarly, Wanyenze et al. conducted a study among WESW where they found that poverty was key in driving the women to engage in sexual risk taking behaviors [34] Therefore, interventions aimed at increasing access to economic resources, including the acquisition of assets among WESW, could help enhance their economic security and build resilience to navigate poverty-associated stressors that would hitherto propel them to further engage in sexual risk behaviors.

Regarding the mediation pathways, we observed a specific indirect effect between asset ownership and sexual risk-taking behaviors through access to medical care. Women who reported higher scores on access to medical care were less likely to engage in sexual risk behaviors. It is possible that women with more assets are more likely to access medical care and consequently engage less in risky behaviors. For instance, Wanyenze’ s study among WESW in Uganda revealed that, due to lack of money, women were unable to access medical services in fear of the expectations of tips by health workers. In addition, some of the respondents mentioned that they couldn’t not afford using condoms unless if they were availed to them for free [34]. Nevertheless, women who may have access to medical services are availed with sufficient information that could protect them from engaging in sexual risk behaviors. A study conducted by Ye and colleagues among female sex workers in Shanghai revealed that access to condoms and health education were strong determinants for consistent condom use among this population [35]. Scorgie and colleagues reported similar results among sex workers in four African Countries, including Uganda, where poverty was identified as a barrier to accessing medical care among WESW and increased their vulnerability to HIV [9]. Therefore, extending medical services to WESW could help reduce the likelihood of engaging in risky sexual behaviors and also lower the rates of HIV among this population.

In addition, our findings revealed a significant relationship between assets and depression, as women who reported having more assets had lower levels of depressive symptoms. However, reduced depressive symptoms were not protective against sexual risk-taking behaviors. WESW with assets may be able to meet their basic life needs, lowering their likelihood of psychological distress. Existing literature has documented a relationship between poverty and mental illness such as depression [10, 36, 37]. For instance, a study by Lee et al. revealed that depression increased women's lifetime exchange of sex for drugs or money, increasing the prevalence of sexual risk behaviors [38]. Similar findings were reported by Musisi and colleagues in their study about primary partnerships in Uganda, where depressive symptoms were associated with higher desires to engage in unprotected sex for financial gains [39]. Given the adverse outcomes of depression resulting from limited resources, efforts should be made to extend economic support to WESW to minimize their chances of engaging in risky behaviors.

### Limitations

This study's findings indicate an indirect relationship between asset ownership and reduced sexual risk-taking behaviors among WESW. However, the interpretation of these results should be made with caution due to some limitations. First, asset ownership was self-reported by participants, which may be prone to social desirability bias. In addition, the study used a cross-sectional design. Therefore, we could not infer causal relationships. Using longitudinal data in future studies would help to ascertain the causal relationships between asset ownership and its impact on sexual risk behaviors among WESW.

## Conclusion

Economic vulnerability influences decisions of WESW to engage in sexual risk behaviors, as they often have multiple customers and engage in unprotected sex for financial gains. Results from this study add to the existing knowledge about the role of poverty or lack of economic resources in women’s decisions to engage in risky sexual behaviors.

### Implication

This study has essential practice, and policy implications as the study findings can inform programs and policies that could reduce engagement in high-risk sexual behaviors among WESW and relatedly the acquisition and spread of HIV through this population. Hence, program implementers should prioritize securing alternative sources of income for WESW, which would eventually reduce their chances of engaging in risky behaviors. In addition, access to healthcare is a strategic target for public health interventions to reduce HIV.

## Data Availability

The datasets analyzed during the current study are available through the corresponding author on reasonable request.
