# Correction: Does asset ownership influence sexual risk‑taking behaviors among women engaged in sex work in Southern Uganda? A mediation analysis

**DOI:** 10.1186/s12905-023-02167-9

**Published:** 2023-01-23

**Authors:** Josephine Nabayinda, Joshua Kiyingi, Samuel Kizito, Edward Nsubuga, Proscovia Nabunya, Ozge Sensoy Bahar, Natasja Magorokosho, Jennifer Nattabi, Susan S. Witte, Fred M. Ssewamala

**Affiliations:** 1 International Center for Child Health and Development (ICHAD), Washington University in St. Louis Brown School, 1 Brookings Drive, St. Louis, MO 63130 USA; 2 Columbia University School of Social Work, 1255 Amsterdam Avenue, New York, NY 10027 USA

**Correction to: BMC Women’s Health (2022) 22:537** https://doi.org/10.1186/s12905-022-02129-7

Following publication of the original article [1], in this article the affiliation details for Author name F. M. Ssewamala were incorrectly given as M. Ssewamala Fred.

The original article has been corrected.
